# Association Between Pandemic Fatigue and Subjective Well-Being: The Indirect Role of Emotional Distress and Moderating Role of Self-Compassion

**DOI:** 10.3389/ijph.2023.1605552

**Published:** 2023-07-11

**Authors:** Qinglu Wu, Peilian Chi, Yan Zhang

**Affiliations:** ^1^ Institute of Advanced Studies in Humanities and Social Sciences, Beijing Normal University, Zhuhai, China; ^2^ Department of Psychology, Faculty of Social Sciences, University of Macau, Macao, Macao SAR, China; ^3^ School of Media and Communication, Shenzhen University, Shenzhen, China

**Keywords:** pandemic fatigue, subjective well-being, emotional distress, self-compassion, conditional process

## Abstract

**Objectives:** As a stressor in the context of COVID-19 pandemic fatigue is associated with well-being. However, how pandemic fatigue is associated with well-being and what protective factors buffer this negative effect are under investigated. Based on the stress process model and emotion regulation theory, the study examined the indirect effect of pandemic fatigue on subjective well-being through emotional distress and the buffering effect of self-compassion.

**Methods:** Data were collected from 1,162 university students (*M*_age_ = 21.61 ± 2.81, female 35.71%) through an online survey. Indirect effect analysis and conditional process analysis were conducted by the SPSS macro PROCESS.

**Results:** Indirect effect of pandemic fatigue on subjective well-being through emotional distress was identified and self-compassion moderated the association between pandemic fatigue and emotional distress. The indirect effect of pandemic fatigue was weaker among participants with high levels of self-compassion than among those with low levels of self-compassion.

**Conclusion:** Pandemic fatigue was negatively associated with subjective well-being through emotional distress at all levels of self-compassion. The findings deepen our understanding of the link between pandemic fatigue and well-being while considering the indirect role of emotional distress and protective function of self-compassion.

## Introduction

Since the end of 2019, the Corona virus disease (COVID-19) pandemic has persisted worldwide and largely affected people’s lives globally. Because of the long-term impacts of the COVID-19 pandemic, the World Health Organization (WHO) [1] highlights the cautiousness on pandemic fatigue, which has raised worldwide concerns. Pandemic fatigue occurs when individuals feel physical and mental/emotional exhaustion due to exposure to COVID-19 information and obeying the behavioral restrictions such as social distancing and quarantine for a long time. People in this situation may be demotivated to maintain the protective behaviors [2, 3]. Negative impacts of pandemic fatigue have been identified, including posttraumatic stress symptoms, information avoidance, sleep problems, and impairment on job contentment [2, 4, 5]. One adverse consequence of fatigue during the pandemic is alleviation of well-being. Negative association between fatigue (e.g., fatigue caused by social media) and well-being (e.g., life satisfaction) has been found in the context of COVID-19 [6–8]. Online fatigue and fatigue caused by social media are negatively associated with psychological well-being and subjective well-being [6, 7].

However, the underlying mechanism between pandemic fatigue and well-being, and whether this negative impact could be buffered by a protective factor, have not been investigated. To address this gap, we propose that emotional distress could work a pathway linking pandemic fatigue and subjective well-being, and self-compassion could function as a protective factor in alleviating the adverse indirect effect of pandemic fatigue on the basis of the stress process model and emotion regulation theory.

The stress process model [9] emphasizes that the initial stressors that individuals experience in daily life could lead to negative consequences by causing secondary stressors, which is also called stress proliferation. Secondary stressors consist of negative experiences (e.g., increased family conflict, symptom barriers and distress) or the damaged resources (e.g., self-efficacy) [10, 11]. The effect of stressors on well-being through causing stress proliferation has been identified in previous studies [12, 13]. Adolescent intimate partner violence is negatively associated with well-being through early adult intimate partner violence and poor health [13]. Following the stress process model, emotional distress could work as a secondary stressor that is caused by the initial stressor of pandemic fatigue, which is further associated with subjective well-being in the context of COVID-19.

Emotional distress has been found to be one adverse consequence of pandemic fatigue in the populations of teachers, university students, and a community sample [14–16]. Individuals who perceive high levels of pandemic fatigue are more likely to be depressed, anxious, and stressed [14]. Epidemic rumination is associated with emotional distress through pandemic fatigue among college students [16]. Emotional distress has been found to play a substantial role in shaping individuals’ subjective well-being and different domains of life (e.g., social relations, functioning, daily activities) [17, 18]. The negative effect of emotional distress (e.g., depressive symptoms, anxiety) on subjective well-being is well-accepted and confirmed in different populations (e.g., college students, schizophrenia patients) [17–19]. For the parents of individuals with developmental disabilities, their caregiving time and severity of behavior problems among their children are associated with subjective well-being through emotional distress [20]. Therefore, individuals suffering information overload and becoming exhausted in the context of COVID-19, they may be more inclined to be depressed and the depressed feelings would make them dissatisfied about their living environment and lives.

Emotion regulation theory highlights the role of personal resources and the approach of emotion regulation strategy in daily life. The importance of self-compassion is emphasized because of its nature as an effective emotional self-regulating strategy and its protective function in buffering the adverse effect of stress [21]. Self-compassion refers to individuals’ positive response toward themselves after experiencing suffering such as life difficulties (e.g., child maltreatment, family strain) and personal inadequacy (e.g., academic failure, personal mistakes). Self-compassion is a multifaceted construct including six components that could be categorized into compassionate self-responding (i.e., self-kindness, common humanity, and mindfulness) and reduced uncompassionate self-responding (i.e., reduced self-judgement, isolation, and over-identification) [22, 23]. Self-compassion is useful to decrease suffering from emotional, cognitive, and attentional aspects [24]. When facing life challenges and suffering, individuals with high levels of self-compassion: a) treat themselves in a kind rather than judgmental way; b) view their suffering as a part of a shared human experience rather than an isolated experience; c) focus on the suffering and deal with adverse thoughts and emotions in a mindful and balanced way rather than in an overly identified manner.

Self-compassion is helpful in lessening the negative impacts of external stress (e.g., racial discrimination, stigma stress) on emotional distress among different populations (e.g., college students, sexual minorities) [21, 25, 26]. Adverse effects of external shame on both depressive and anxiety symptoms are weaker among university students with high levels of self-compassion than among those with low levels of self-compassion [27]. Positive associations between cyberbullying victimization and emotional distress (e.g., depressive symptoms, anxiety) are found among adolescents with low levels of self-compassion. For the adolescents with high levels of self-compassion, the associations are non-significant [28]. Thus, it is possible that self-compassion could buffer the effect of pandemic fatigue on emotional distress and their association is weaker or even absent among the individuals with high levels of self-compassion.

Supported by the stress process model and emotion regulation theory, the present study examined the indirect effect of pandemic fatigue on subjective well-being through emotional distress and the moderating role self-compassion in attenuating the association between pandemic fatigue and emotional distress.

## Methods

### Participants

Data were from a project on the effect of COVID-19 pandemic on psychological adjustment (e.g., mental health, well-being) among Chinese university students. University students (i.e., undergraduates and postgraduates) aged 18 years or above from Beijing Normal University were recruited to participate the study. Data were collected in April 2022. Participants were informed of the research purpose, confidentiality, and their rights to voluntary participation. Valid participants received a small monetary reward (30 RMB, approximately US $ 4.5). The project was approved by the research ethics committee of the School of Social Development and Public Policy at Beijing Normal University before data collection.

A total of 1,317 qualified participants completed the online survey. After removing cases with duplicated responses (*n* = 37) and those that failed the attention check (*n* = 118), the final sample size was 1,162 (35.7% male). The average age of the participants was 21.61 (*SD* = 2.81). The percentages of undergraduates and postgraduates were 65.2% and 34.8% respectively. The educational attainment of 22% of the participants was high school or below. The household monthly income was 19,811.49 RMB (*SD* = 48045.29, approximately US $ 2,968.90).

### Measures

Pandemic fatigue is the independent variable in the present study. It was measured by the pandemic fatigue scale developed by Lilleholt [29]. This scale has six items, using a 7-point Likert scale ranging from 1 (*strongly disagree*) to 7 (*strongly agree*). Sample items are “I am tired of all the COVID-19 discussions in TV shows, newspapers, and radio programs, etc.” and “I am tired of restraining myself to save those who are most vulnerable to COVID-19.” A total mean score was calculated by dividing the total score by the number of items. A high mean value represented a higher level of pandemic fatigue. The Cronbach’s *α* of the scale in the present study was 0.91.

Emotional distress is the mediator in the hypothesized model. The Depression subscale of the Depression-Anxiety-Stress Scale developed by Lovibond [30] was used to measure emotional distress. Seven items were rated on a 4-point scale ranging from 1 (*did not apply to me at all*) to 4 (*applied to me very much*). Sample items are “I felt that life was meaningless.” and “I was unable to become enthusiastic about anything.” A total mean score was calculated by dividing the total score by the number of items. A high mean value represented a higher level of emotional distress. The Cronbach’s *α* of this scale in the present study was 0.89.

Self-compassion is the moderator in the hypothesized model. Self-compassion was assessed by the Self-Compassion Scale-Short Form constructed by Raes (SCS-SF) [31]. All 12 items were rated on a 5-point Likert scale ranging from 1 (*almost never*) to 5 (*almost always*). Sample items are “When I’m going through a very hard time, I give myself the caring and tenderness I need.” and “When something upsets me I try to keep my emotions in balance.” A total mean score was calculated by dividing the total score by the number of items. A high mean value represented a higher level of self-compassion. The Cronbach’s *α* of SCS-SF in the present study was 0.80.

Subjective well-being is the dependent variable in the present study. Subjective well-being was assessed by the Satisfaction With Life Scale (SWLS) developed by Diener [32]. This scale consists of five items, which were rated on a 7-point Likert scale ranging from 1 (*strongly disagree*) to 7 (*strongly agree*). Sample items are “I am satisfied with my life.” and “The conditions of my life are excellent.” A total mean score was calculated by dividing the total score by the number of items. A high mean value indicated a higher level of subjective well-being. Cronbach’s α of the SWLS in the present study was 0.87.

### Data Analysis

**FIGURE 1:**
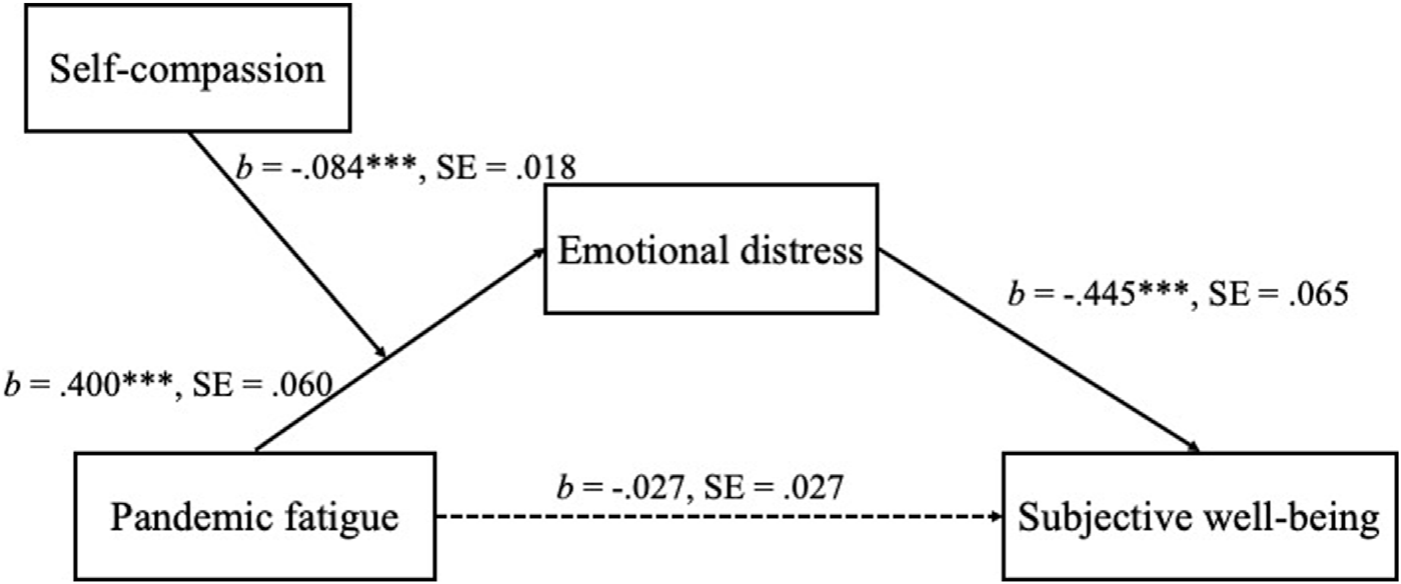
Specific paths and unstandardized coefficients for the hypothesized model (China, 2022). Note. Demographic variables and whether participants or people they know have been infected with COVID-19 were controlled. *** *p* < 0.001.

Descriptive statistics (i.e., mean, standard deviation, ranges of the scores, internal consistency coefficients, Pearson correlations among the key variables) were conducted by SPSS (version 24). Indirect effect and conditional indirect effect (moderating effect of self-compassion) were found by conducting indirect effect analysis and conditional process analysis (for the hypothesized model, see Figure 1), which were examined by the SPSS macro PROCESS (Model 4 and Model 7, respectively) developed by Hayes [33]. A resampling approach with bias-corrected bootstrapping (5,000 times) was used to address the sampling distribution of the indirect effect and moderating effect. Bias-corrected 95% confidence intervals (CIs) were used to assess the significance of the indirect effects and moderating effect. Pandemic fatigue and self-compassion were mean-centered for analyzing the moderating effect of self-compassion. If the interaction term of mean-centered pandemic fatigue and self-compassion was significant, simple slope analysis was performed to further examine the conditional slope of the association between pandemic fatigue and emotional distress at high (one standard deviation above the mean) and low (one standard deviation below the mean) levels. Moreover, a conditional indirect effect was also analyzed. Age, sex, educational background, family monthly income, and whether participants or people they know have been infected with COVID-19 were controlled for when model was examined.

## Results

Table 1 demonstrates the basic information (i.e., Pearson correlations, means, SDs) of the key variables. Pandemic fatigue was positively associated with emotional distress and both of them were negatively associated with self-compassion and subjective well-being. Self-compassion was positively associated with subjective well-being.

**TABLE 1:** Descriptive statistics and correlations among the key variables (China, 2022).

	1	2	3	4
1. Pandemic fatigue	—			
2. Emotional distress	0.43***	—		
3. Self-compassion	−0.32***	−0.50***	—	
4. Subjective well-being	−0.11	−0.22***	0.41***	—
Mean	3.18	1.59	3.35	4.46
Standard deviation	1.48	0.62	0.54	1.27
Range	1–7	1–4	1.5–5	1–7

Note. *** *p* < 0.001.

Results of indirect effect analysis showed that pandemic fatigue was indirectly associated with subjective well-being through emotional distress (*b* = −0.078, SE = 0.014, 95% CI [−0.106, −0.052]). Pandemic fatigue was positively associated with emotional distress (*b* = 0.175, SE = 0.011, 95% CI [0.153, 0.197]) and emotional distress was negatively associated with subjective well-being (*b* = −0.445, SE = 0.065, 95% CI [−0.571, −0.319]). The direct effect of pandemic fatigue on subjective well-being was not significant (*b* = −0.027, SE = 0.027, 95% CI [−0.080, 0.026]).

Table 2 displays the results of the conditional process analysis. When self-compassion was entered as a moderator in the association between pandemic fatigue and emotional distress, the interaction between pandemic fatigue and self-compassion had a significant effect on emotional distress (*b* = −0.084, SE = 0.018, 95% CI [−0.119, −0.050]). This result suggests that self-compassion moderated the association between pandemic fatigue and emotional distress. Figure 1 presents the specific paths and coefficients for the hypothesized model.

**TABLE 2:** Multivariate regression testing the hypothesized model (China, 2022).

Predictor	Outcome 1: Emotional distress				Outcome 2: Subjective well-being			
	B	SE	t	95% CI	B	SE	t	95% CI
Age	0.019	0.008	2.421*	[0.004, 0.034]	0.064	0.019	3.389***	[0.027, 0.101]
Sex	−0.034	0.031	−1.084	[−0.095, 0.028]	−0.244	0.077	−3.190**	[−0.394, −0.094]
Educational background	−0.105	0.040	−2.617**	[−0.183, −0.026]	−0.209	0.098	−2.133*	[−0.401, −0.017]
Family monthly income	−0.011	0.015	−0.751	[−0.041, 0.018]	0.060	0.037	1.619	[−0.013, 0.133]
Infected with COVID-19	−0.164	0.046	−3.545***	[−0.255, −0.073]	−0.204	0.114	−1.792	[−0.427, 0.019]
PF	0.118	0.011	11.135***	[0.097, 0.139]	−0.027	0.027	−1.001	[−0.080, 0.026]
SC	−0.488	0.029	−16.845***	[−0.544, −0.431]				
PF × SC	−0.084	0.018	−4.764***	[−0.119, −0.050]				
ED					−0.445	0.065	−6.903***	[−0.571, −0.319]
*R* ^2^	0.356				0.078			
F	79.582***				13.915***			

Note. PF, pandemic fatigue; SC, self-compassion; ED, emotional distress, * *p* < 0.05, ** *p* < 0.01, *** *p* < 0.001.

**FIGURE 2:**
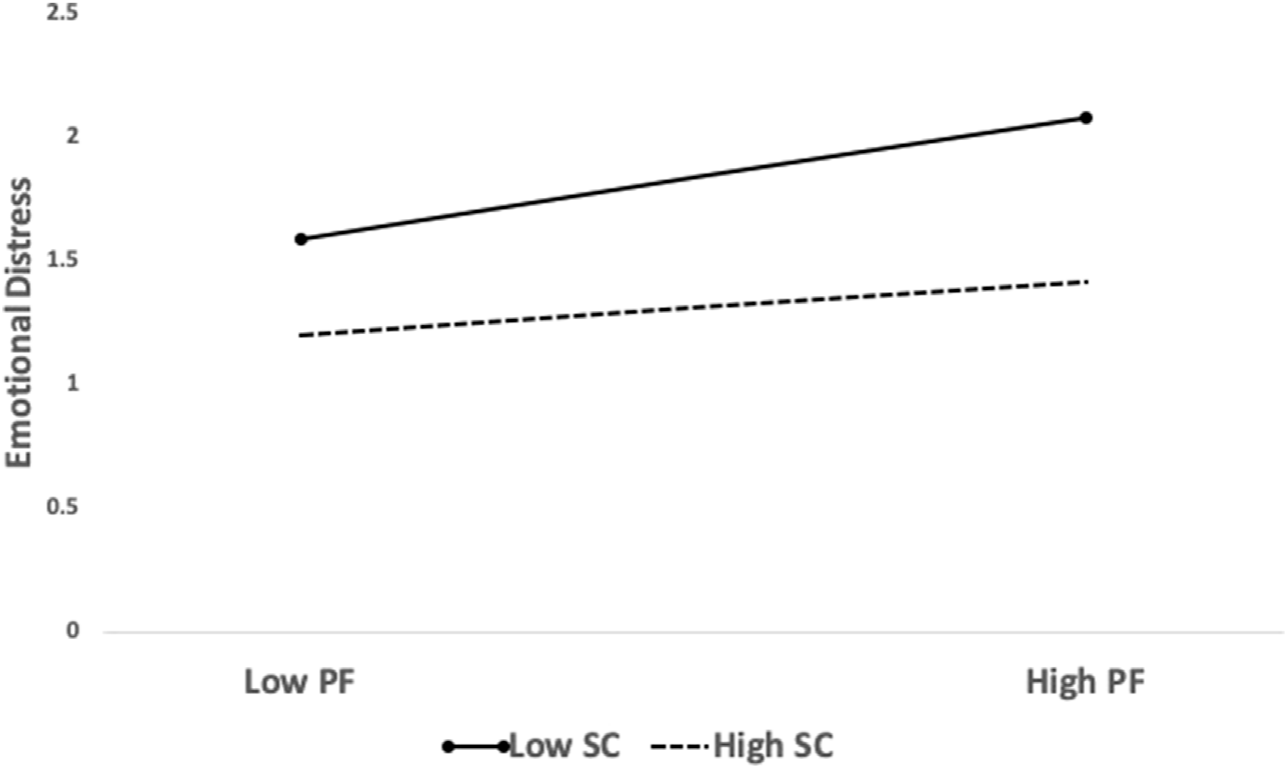
Plots of the interaction of pandemic fatigue and self-compassion on emotional distress (China, 2022).

Simple slope analysis was employed to investigate the relationship between pandemic fatigue and emotional distress at high (one SD above the mean; *N* = 158) and low (one SD below the mean; *N* = 188) levels of self-compassion. The positive association between pandemic fatigue and emotional distress and the negative indirect effect of pandemic fatigue on subjective well-being through emotional distress were identified at all self-compassion levels. The association between pandemic fatigue and emotional distress was positively significant (low level: *b* = 0.163, SE = 0.014, 95% CI [0.136, 0.191]; high level: *b* = 0.072, SE = 0.014, 95% CI [0.044, 0.100]). With the increase of self-compassion, the predictive effect of pandemic fatigue on emotional distress decreased (see Figure 2). Moreover, the indirect effect of pandemic fatigue on subjective well-being through emotional distress was statistically significant at both self-compassion levels (low level: *b* = −0.073, SE = 0.014, 95% CI [−0.101, −0.048]; high level: *b* = −0.032, SE = 0.008, 95% CI [−0.049, −0.018]). The indirect effect of pandemic fatigue became weaker as the level of self-compassion increased.

## Discussion

The present study examined the indirect effect of pandemic fatigue on subjective well-being through emotional distress and the buffering effect of self-compassion in this association. Pandemic fatigue was indirectly associated with subjective well-being through emotional distress. In addition, self-compassion moderated the association between pandemic fatigue and emotional distress. The present study was conducted in the period during which Chinese people faced COVID-19 information overload and obeyed the travel and behavioral restriction. People living in the context of COVID-19 need to notice the pandemic information when arranging their schedule and strictly obey the restrictions (e.g., lockdown, social distancing, quarantine), which occupies a lot of attention, energy, and psychological resources. Individuals who spend a lot of energy and resources to cope with COVID-19 related stress are more likely to be fatigued. Exhaustion caused by exposure to COVID-19 pandemic information from social media and behavioral restrictions usually leaves individuals tired [29], which makes them inclined to suffer emotional problems like emotional distress. Individuals who feel more fatigue in the COVID-19 pandemic are more likely to be depressed, anxious, and stressed [14, 16]. In the context of COVID-19, emotional distress would further reduce their subjective well-being. Depressed people feel hopeless towards the future, feel that life is meaningless, and lack enthusiasm about things around them. Thus, they are inclined to be less satisfied with life and give it a low evaluation [18, 19].

One noteworthy finding is that self-compassion buffered the indirect effect of pandemic fatigue on subjective well-being through emotional distress. Specifically, self-compassion alleviated the association between pandemic fatigue and emotional distress. This association was weaker among the individuals with high levels of self-compassion than among those people with low levels of self-compassion. This finding is in line with previous findings that self-compassion functions as an effective emotion regulation strategy in attenuating the adverse effect of different stressors on emotional distress [25, 27, 34]. Stressors (e.g., shape overvaluation, academic burn-out) are associated with emotional distress (e.g., depressive symptoms, anxiety) among the people with low levels of self-compassion, and these associations are absent or weaker among groups with high levels of self-compassion [28, 35, 36]. Our findings suggest that self compassion serves a protective role by way of activating self-soothing systems, which functions well for alleviating stress and sense of threat [37]. It is well accepted that individuals with high self-compassion may treat themselves in a friendly way, view their suffering from stress as the negative experiences that are shared by human beings, and keep balance in negative thoughts and emotion when they face stress and life difficulties [21, 26]. Individuals in the context of COVID-19 with high self-compassion may view the stressful experiences (e.g., pandemic fatigue) related to COVID-19 pandemic as suffering that is shared by the many people around them. Thus, they may feel relieved from the suffering and negative experiences. In addition, these people could treat themselves in a kind way during this tough time and find balance in negative thoughts and emotions, which is helpful in alleviating emotional distress associated with external stress.

The present study has insightful implications. To our knowledge, the present study is the first to investigate the emotional pathway between pandemic fatigue and subjective well-being in association with the buffering effect of a protective factor. Exposure to COVID-19 pandemic information from social media and behavioral restrictions make individuals physically and mentally exhausted. These people are more vulnerable to the emotional problems and distress, which further damages their satisfaction with life. This finding extends the implications of the stress process model in the context of COVID-19. Pandemic fatigue was the initial stressor that was associated with subjective well-being through the secondary stressor of emotional distress. In addition, our findings provide new understanding and supplement the roles of protective factors (e.g., mastery, self-esteem, schedule control) in weakening the link between stress and outcomes [38–40] during COVID-19 pandemic. This indirect emotional pathway was buffered by self-compassion, which functioned as a protective factor in reducing the negative effect of pandemic fatigue on emotional distress. Our finding confirms that as an effective emotional self-regulation strategy, self-compassion is helpful in alleviating the suffering from the external stress of pandemic fatigue in the context of COVID-19. Moreover, our findings provide insights into the practice on fatigue prevention and treatment of emotional distress in the context of COVID-19. Interventions (e.g., Mindfulness and Compassion-based Intervention) could be applied to reduce fatigue and emotional distress and improve self-compassion in the context of COVID-19 [41, 42].

### Limitations and Future Research

The present study has several limitations. First, the cross-sectional design of the current study limits the investigation of the causal relationships among pandemic fatigue, emotional distress, and subjective well-being. The association between pandemic fatigue and subjective well-being, the mediating effect of emotional distress, and the buffering role of self-compassion should be examined in the future research with a longitudinal design. Second, the self-reported and retrospective nature of the data may lead to response and recall biases. Other approaches of data collection such as assessment of physical fatigue (e.g., hand grip strength) [43] could be used in future studies. Third, emotional distress was measured by a single indicator of depressive symptoms. Indirect effects including multiple emotional distress (e.g., depressive symptoms, anxious symptoms) may be different due to the distinctions of the indicators of emotional distress. To examine potential indirect effects of pandemic fatigue on subjective well-being, multiple emotional distress factors should be measured and added to the hypothesized model in the future study. Fourth, the protective function of self-compassion was examined in a survey study, which could not examine its effectiveness in the treatment. Future research could investigate the buffering effect of self-compassion in an intervention study with a randomized controlled trial design [44].

### Conclusion

In the present study, pandemic fatigue was found to be associated with subjective well-being through emotional distress among university students. In addition, self-compassion alleviated the negative effect of pandemic fatigue on well-being by weakening the association between pandemic fatigue and emotional distress. Future studies and interventions aimed at reducing the adverse effect of COVID-19 pandemic could focus on the emotional pathway in the link between pandemic fatigue and outcomes, and the protective function of emotion regulation strategy.

## AUTHOR’S NOTE

This manuscript is not under review elsewhere and the results have not been published previously or accepted for publication. This manuscript has been seen and approved by all authors.

## Data Availability

All data are available and can be obtained by emailing: qinglu-wu@hotmail.com.
